# Multi-omics analysis reveals lysosome-associated molecular subtype characterization and prognostic modeling system in lung adenocarcinoma

**DOI:** 10.7150/jca.105351

**Published:** 2025-02-18

**Authors:** Zhanmei Wang, Yan Wang, Jinxiang Wang

**Affiliations:** 1 Department of Oncology, Qilu Hospital, Cheeloo College Medicine, Shandong University, Qingdao, 266000, China.; 2 Department of infectious diseases, Qilu Hospital, Cheeloo College of Medicine, Shandong University, Qingdao, 266000, China.; 3 Department of Respiratory and Critical Care Medicine, Qilu Hospital, Cheeloo College Medicine, Shandong University, Qingdao, 266000, China.

**Keywords:** lung adenocarcinoma, lysosome, molecular subtype, single-cell sequencing analysis, SLC2A1

## Abstract

**Background:** Lung adenocarcinoma (LUAD) poses a significant challenge in current treatments due to its high recurrence and metastasis rates. Despite preliminary evidence suggesting the role of lysosomes in LUAD, it remains unclear whether lysosome-related functions can be effectively used for risk stratification of LUAD patients and involved lysosome-related functional targets are still needed to be explored.

**Method:** An integrated analysis of TCGA and GEO databases was conducted to explore the potential role of lysosome-related genes (LRGs) in LUAD. Unsupervised consensus clustering analysis was utilized to explore the LRG molecular subtypes in LUAD. ESTIMATE and ssGSEA algorithms were performed to evaluate the immune infiltration characterization of LUAD samples. LASSO-univariate and multivariate Cox analysis were used to construct the LRG score model. Single-cell sequencing analysis was performed to reveal the distribution characteristics in different cell subpopulations of selected LRGs. *In vitro* experiments including western blotting, PCR, colony formation assays, and Transwell assays were used to verify the expression and biological functions of the selected target in LUAD.

**Results:** Through multi-omics integration analysis algorithms, we successfully developed a prognostic risk stratification system based on LRG scoring in LUAD and constructed a nomogram diagnostic model. Various bioinformatics analyses indicated the potential clinical value of the LRG scoring system. Single-cell sequencing analysis further revealed the composition of cell subpopulations and the expression characteristics of prognostic signatures. SLC2A1, one of the selected targets, was validated through *in vitro* experiments to regulate the proliferation and migration of LUAD cells, thereby confirming the reliability of the bioinformatics results.

**Conclusion:** Our results demonstrate that effective risk stratification of LUAD patients can be achieved through LRGs by multi-omics analysis integration. Furthermore, we validated key prognostic targets* in vitro*, providing new ideas for future clinical treatment.

## Introduction

As one of the leading causes of cancer-related mortality globally, lung adenocarcinoma (LUAD) presents a significant challenge due to its lack of specific peripheral symptoms; approximately 50%-60% of patients are diagnosed with distant metastasis at initial presentation 1. Moreover, LUAD is characterized by a high likelihood of recurrence, posing a challenge in current therapeutic approaches 1, 2. Further elucidating the potential molecular mechanisms guiding tumor progression and identifying novel intervention targets holds crucial clinical significance 3, 4.

Lysosomes, acidic intracellular organelles containing active hydrolytic enzymes and specific membrane proteins, are ubiquitous in animal cells except for red blood cells 5. Lysosomes not only uptake extracellular and intracellular materials through endocytic and autophagic pathways but also secrete their contents through fusion with the plasma membrane 6. The bidirectional transport function of lysosomes facilitates various biological processes, including cell death, signal transduction, immune responses, and stress responses 7. Additionally, lysosomes play a crucial role in mediating cell apoptosis and necrosis through a process known as lysosomal membrane permeabilization (LMP), thereby serving as key regulators in cell death signaling pathways 8.

There is evidence indicates alterations in lysosomal morphology across all cancer types 9. Soluble hydrolytic enzymes contained within lysosomes are pivotal for tumorigenesis processes 9. Among the extensively studied lysosomal hydrolytic enzymes, cytoplasmic cathepsin proteases can inhibit tumor growth by 10 activating intrinsic apoptotic pathways 11. Conversely, extracellular cathepsin proteases promote tumor growth by degrading the basement membrane and activating other pro-tumorigenic proteins 12. The three subtypes of cathepsin proteases have been implicated in cancer progression and metastasis 13-15. The impact of lysosomal dysfunction on tumor chemoresistance has also drawn increased attention to lysosomes 16. Moreover, through complex interactions with various cancer-related signaling pathways, lysosome participates in mechanisms facilitating cancer evasion of immune surveillance and cell apoptosis 7. Recently, therapies that target the regulation of lysosomes and associated autophagic processes to influence tumor progression have garnered increasing attention 10. By disrupting lysosomal homeostasis, lysosome-related inhibitors can selectively inhibit tumor cell proliferation without adverse effects on normal cells, thereby demonstrating significant potential for clinical applications 17. Advances in nanomaterials and related drug delivery systems have further enhanced the feasibility of targeting lysosomes in cancer therapy 18, 19. However, the role of lysosomes in LUAD remains incompletely understood, and the potential molecular mechanisms of lysosomal involvement in cell apoptosis and associated genes in LUAD have yet to be elucidated.

This study identified lysosome-related genes (LRGs) associated with LUAD through analysis of TCGA and GEO databases, subsequently recognizing LRG-related molecular subtypes. Subsequently, we constructed an LRG scoring system and validated its independent prognostic assessment capability. Single-cell sequencing analysis further revealed the composition of cell subpopulations and the expression characteristics of prognostic signatures. Furthermore, among the screened targets, SLC2A1 was validated through *in vitro* experiments for its ability to regulate LUAD cell proliferation and migration, thereby confirming the reliability of the bioinformatics results. Our findings provide evidence for the involvement of lysosomes in the development of LUAD and identify potential intervention targets.

## Materials and Methods

### Acquisition and preprocessing of transcriptomic gene features in LUAD samples

Utilizing the TCGA and GEO databases, we acquired comprehensive genomic gene expression data of both LUAD samples and normal tissue samples along with clinical baseline information. Based on the TCGA database, we extracted 507 primary LUAD samples and 59 paracancerous normal tissue samples for subsequent analysis. Among them, LUAD samples lacking survival time or survival status and containing other disease types were excluded in this study. Additionally, we downloaded raw data on mutation burden characteristics (TMB) and copy number variations (CNV) of LUAD samples from the TCGA database. Employing Perl scripting within the Perl language environment, we executed Perl scripts to annotate the transcriptomic features of samples in the TCGA database using the human genome annotation script. Moreover, the GSE72094 dataset was obtained from the GEO database (GPL15048) and processed using Perl scripting to align and annotate gene features according to the annotation file of the gene platform. Notably, after excluding LUAD samples with missing survival information, we extracted a total of 397 primary LUAD samples for subsequent analysis. To eliminate batch effects between the transcriptomic data from the two databases, we used the "sva" R package to clean and normalize the data for analysis. After excluding samples lacking clinical survival time and prognosis, a total of 904 LUAD samples were collected for final data analysis, comprising 507 primary LUAD samples from the TCGA database and 397 primary LUAD samples from the GSE72094 dataset (Table 1).

### Identification of Lysosome-Related Gene (LRG) signatures and recognition of LRG-related molecular subtypes

In this study, lysosome-related genes (LRGs) were obtained from the MSigDB database (www.gsea-msigdb.org/), where a total of 163 LRG genes were collected for subsequent analysis. Using the "limma" script, we extracted and analyzed the differential expression of LRG gene signatures between normal samples and LUAD samples. Subsequently, the "pheatmap" script was employed for visual analysis, with a differential screening threshold set at |FC|>2 and P (adjust)<0.05. Based on these differentially expressed LRG gene signatures, we utilized the "ConsensusClusterPlus" R package to explore and identify molecular subtypes of LUAD. Using the K-means clustering algorithm, we calculated model parameters for different cluster numbers (k=2-10). Utilizing the optimal parameters of the model (Delta area and CDF), LUAD samples were categorized into different LRG molecular subtype subgroups. Additionally, the "ggplot2" script was used to analyze the PCA pattern of LRG molecular subtypes, assessing the reliability and accuracy of the consensus clustering analysis.

### Evaluation analysis of immune microenvironment infiltration features

Based on sample-based gene transcriptomic features, we employed the "ESTIMATE" algorithm to assess immune infiltration characteristics among samples, and thoroughly evaluated four immune-related indicators, including immune score, stromal score, ESTIMATE score, and tumor purity. Additionally, leveraging gene markers for 23 immune cells, we utilized the "limma" script to extract the gene expression levels of immune cells for each sample, and conducted ssGSEA analysis via the "GSVA" script to evaluate the immune cell infiltration proportions in each sample.

### Analysis of potential regulatory mechanisms of LRG molecular subtypes

Utilizing the "limma" script, with threshold conditions set as |fold change| > 2 and p.adjust < 0.05, we computed the differential gene expression status between LRG molecular subtypes and identified the genes comprising the differentially expressed gene signature. Employing the "clusterProfiler" script, we assessed Gene Ontology (GO) and Kyoto Encyclopedia of Genes and Genomes (KEGG) entries of differentially expressed genes between LRG molecular subtypes to explore potential molecular mechanisms. Furthermore, using the KEGG reference gene signature "c2.cp.kegg.v7.2.symbols," we conducted "GSVA" analysis to evaluate the differentially regulated KEGG pathways between LRG molecular subtypes. Differential gene signatures between LRG molecular subtypes were extracted using the "limma" script, and gene subtype classification related to LRG molecular subtypes was performed based on "ConsensusClusterPlus." Utilizing the optimal classification ratio among cluster numbers (k = 2-10), LUAD samples were categorized into distinct gene subtypes. Integrating clinical baseline data of LUAD and classification of different subtypes, we employed "pheatmap" for visual analysis of the differentially expressed gene signatures.

### Construction of LRG scoring system and analysis of clinical pathological subgroups

Integrating the expression profiles of differentially expressed gene signatures with clinical survival data of LUAD samples, we utilized the "survival" and "glmnet" scripts to analyze variables with prognostic value and constructed a LASSO model to further validate feature variables. Through multivariate Cox analysis, we computed and obtained variables with independent prognostic value, along with calculating the risk scores for each variable. Based on the risk scores of variables and expression profiles, we established an LRG scoring system to classify LUAD samples into risk categories: LRG score = (0.147 × ANLN) + (-0.116 × IRX2) + (0.163 × SLC2A1). Using the cutoff value of clinical survival for LUAD samples, we divided the LUAD samples into low LRG score group and high LRG score group. Employing the "ggalluvial" script, we analyzed LRG molecular subtypes, gene subtypes, and LRG scores using the ggalluvial model. With the "caret" R package, LUAD samples were split into training and validation sets at a ratio of 6:4. Based on the optimal cutoff value of clinical outcomes for LUAD, LUAD samples in the two independent sets were classified into LRG score subgroups. Using the "survival" script, we statistically analyzed the clinical prognostic status of LUAD in the two independent sets, validating the accuracy and precision of the LRG scoring system in predicting clinical prognosis of LUAD samples. Finally, integrating the clinical baseline data of LUAD samples with LRG scores, we utilized the "survival" script to explore the clinical survival status of LRG score subgroups among different clinical-pathological features.

### LRG score independent prognostic assessment and nomogram model construction

Combining the clinical pathological data of LUAD samples with LRG scores, we performed univariate and multivariate Cox analyses within the "survival" environment to assess the independent prognostic value of different clinical pathological variables and LRG scores for LUAD. Using the "survivalROC" script, we plotted ROC curves for LUAD samples at 1-year, 3-year, and 5-year intervals and calculated the AUC values. Based on the clinical pathological features of LUAD samples and LRG scores, we utilized the "caret" R package to construct a nomogram model to predict the survival probability of LUAD samples at 1-year, 3-year, and 5-year intervals.

### Somatic mutation features, immune therapy response, and drug sensitivity prediction

In the Perl language environment, we extracted the TMB (MAF Format) scores from LUAD sample files to assess the TMB scores of LRG scoring subgroups. Using the "maftools" script, we analyzed the frequency characteristics of somatic mutations within LRG scoring subgroups and generated waterfall plots. The LUAD IPS files were obtained from the TCIA database (https://tcia.at/home) and subjected to classification analysis based on LRG scoring subgroups. Utilizing the GDSC database, we analyzed potential small molecule compounds that may exhibit responses within LRG scoring subgroups based on the gene transcriptomic features of LUAD samples.

### Acquisition and preprocessing of single-cell RNA sequencing data

In this study, single-cell RNA sequencing (scRNA-seq) data of lung adenocarcinoma were obtained from the GEO database, with the dataset accession number GSE223923. Data processing was performed using the "Seurat" R package (v4.0.5) in R, with the data stored in rds format. Initially, quality control was applied to each sample, filtering out cells with fewer than 200 or more than 2,500 detected genes, as well as those with more than 10% mitochondrial gene expression, to ensure high data quality. The gene expression matrix was then normalized using the “NormalizeData” function, scaling the gene expression of each cell to a total count of 10,000. To integrate data across multiple samples, the “FindIntegrationAnchors” function was employed to identify anchors, followed by the “IntegrateData” function for data integration. Principal component analysis (PCA) was performed on highly variable genes for dimensionality reduction, with the top 20 principal components used to construct a K-nearest neighbor (KNN) graph to capture similarities between cells. Clustering of cells was conducted using the “FindNeighbors” and “FindClusters” functions, which identified distinct cellular populations. Based on the clustering results, differential gene expression analysis was performed for each cell cluster using the “FindAllMarkers” function, and the populations were annotated with the “singleR” annotation algorithm. Additionally, t-SNE (t-distributed stochastic neighbor embedding) and UMAP (uniform manifold approximation and projection) algorithms were used for two-dimensional visualization of the cell clusters, providing a clear representation of cellular heterogeneity.

### Cell culture

Human LUAD cell lines H1299 and A549 were purchased from the American Type Culture Collection (ATCC). The above-mentioned cells were cultured in 1640 medium containing 10% fetal bovine serum and 1% penicillin-streptomycin. All cells were maintained in a sterile humidified incubator at 37°C and 5% CO2. Unique short tandem repeat (STR) analyses were performed periodically to confirm the authenticity of cell lines. Regular mycoplasma testing was conducted to verify that all cells were free of mycoplasma contamination.

### Western blot analysis

Cell collection and lysis were performed on ice using RIPA buffer (Beyotime, P0013B) containing 1% PMSF. Subsequently, the lysates were centrifuged at 12,000 g at 4°C for 20 minutes. The protein concentration in each sample was determined using the Bicinchoninic Acid (BCA) protein assay. Next, proteins were heated at 95°C for 10 minutes in loading buffer, then loaded onto 10% polyacrylamide gels for electrophoresis. Electrophoresis was conducted at a constant voltage of 120 V in Tris-glycine buffer for 1 hour. The proteins were then transferred from the gel to a polyvinylidene difluoride (PVDF) membrane (Millipore, IPVH00010). The membrane was blocked in TBST buffer containing 5% skim milk at room temperature for 1 hour. Subsequently, the membrane was incubated with anti-SLC2A1 (1:1000; Abcam, ab280797) and anti-ATCB (1:5000; ABclonal, AC026) antibodies overnight at 4°C, followed by incubation with corresponding horseradish peroxidase (HRP)-conjugated secondary antibodies at room temperature for 1 hour. Protein bands were visualized using the SuperPico ECL luminescence reagent (Vazyme, E422-01), and band intensities were quantitatively analyzed using Image J software.

### Reverse transcription, Real-Time Qualitative PCR (RT-PCR)

Total RNA extraction and subsequent reverse transcription experiments were performed according to the manufacturer's instructions of the RNA purification kit (Fastagen, 220011) and the RevertAid First Strand cDNA Synthesis kit (Thermo Fisher, K1622), respectively. RT-PCR was performed using the Hieff® qPCR SYBR Green Master Mix (TEASEN, 11201ES03) on the CFX96™ optical module (BIO-RAD). The primer sequences used were as follows: forward, TGAGCATCGTGGCCATCTTT; reverse, CCGGAAGCGATCTCATCGAA.

### Colony-formation assay

Cells were seeded in six-well plates at a density of 1000 cells per well and cultured for 1-2 weeks at 37°C and 5% CO2 until visible cell colonies formed. The culture medium was then removed, and the cells were fixed with methanol for 30 minutes. Subsequently, the cells were stained with 0.1% crystal violet solution for 30 minutes. After staining, the dye solution was gently washed off with water, and the colonies were air-dried before photography. Colonies with a diameter greater than 0.1 mm were counted.

### Transwell assay

Cells were digested, centrifuged, and resuspended in serum-free culture medium for cell counting. A total of 100 μL of cell suspension containing 100,000 cells was added to the upper chamber. Then, 600 μL of complete culture medium was added to the lower chamber of a 24-well plate. The cells were incubated in a cell culture incubator for 48 hours. Subsequently, the cells were fixed with methanol for 30 minutes, then stained with 0.5% crystal violet solution for 30 minutes. After washing the upper chamber with PBS, the dye on the upper surface was gently wiped off with a cotton swab. The cells were then observed, photographed, and counted under an inverted microscope.

### Statistical analysis

In both R and Perl language environments, we preprocessed data from LUAD samples and conducted visualization analysis using R packages. Pearson correlation algorithm was employed to calculate the correlation between LRG scores and 23 types of immune cells. For statistical analysis between two groups of data, we utilized the Wilcoxon rank-sum test; for multiple group comparisons, the One-way ANOVA test was employed. In this study, all statistical differences (P values) were subjected to multiple testing corrections, and those with P < 0.05 were considered statistically significant. Significance levels are denoted as follows: *p < 0.05; **p < 0.01; ***p < 0.001; ****p < 0.0001.

## Results

### Differential analysis and mutation feature prediction of LRG gene signature in LUAD

**Figure 1:**
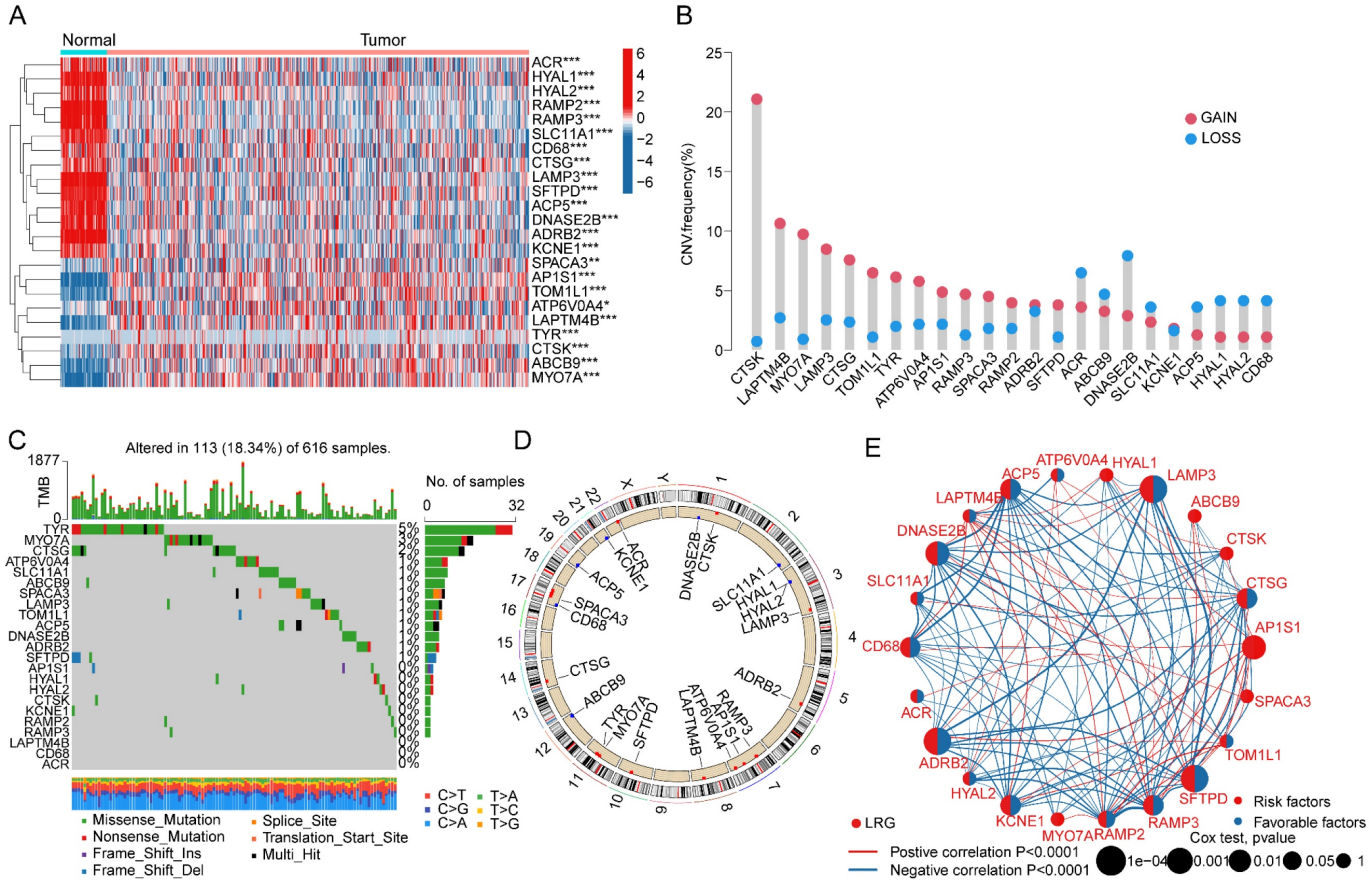
** Differential Expression Analysis and Mutation Landscape Prediction of LRG Gene Signatures in LUAD. (A)** Differential analysis of LRG gene signatures between normal tissues and LUAD tissues. Selection criteria: |FC| > 2, p.adjust < 0.05. **(B)** Calculation of copy number variation coefficients for differentially expressed LRGs. **(C)** Mutation landscape characteristics of LRG gene signatures in LUAD. **(D)** Chromosomal distribution of LRG gene signatures. **(E)** Prognostic evaluation of LRG gene signatures in LUAD. HR > 1 represents a risk factor, while HR < 1 represents a benefit factor.

In this study, a total of 163 lysosome-related gene signatures (LRGs) were included to explore their potential biological functions and mechanisms in LUAD. Using the "limma" script, we analyzed the differential expression of LRG gene signatures between normal tissues and LUAD. The results indicated significant expression differences for 23 LRGs between normal tissues and LUAD tissues, with 14 LRG gene signatures showing significantly increased expression in normal tissues and 9 LRG gene signatures showing significant overexpression in LUAD tissues (Figure 1A, |FC| > 2, p < 0.05). To better understand the potential roles of these differentially expressed LRGs in LUAD, we investigated the copy number variation (CNV) and mutation burden characteristics of LRGs. CNV prediction results suggested significant CNV amplification for CTSK, LAPTM4B, MYO7A, LAMP3, CTSG, TOM1L1, TYR, ATP6V0A4, AP1S1, RAMP3, SPACA3, SFTPD, and RAMP2 in LUAD, while ACR, ABCB9, DNASE2B, SLC11A1, ACP5, HYAL1, HYAL2, and CD68 exhibited significant CNV deletion (Figure 1B). Mutation burden characteristics indicated that among 616 LUAD samples, 113 samples had mutations in LRG gene signatures (18.34%), with mutation frequencies of 5%, 3%, and 2% for TYR, MYO7A, and CTSG, respectively (Figure 1C). Additionally, the chromosomal co-localization map illustrated the distribution of these LRG gene signatures on different chromosomes, suggesting their potential genetic relevance to LUAD (Figure 1D). Furthermore, network analysis results further demonstrated the potential relationship between LRG gene signatures and clinical survival prognosis in LUAD. Among the 23 differential LRGs, 10 LRG gene signatures were associated with survival benefits in LUAD, including CD68, ADRB2, ACP5, DNASE2B, RAMP3, LAMP3, KCNE1, RAMP2, CTSG, and SFTPD (HR < 1, P < 0.05), while AP1S1 was associated with poor prognosis in LUAD (HR > 1, P < 0.05) (Figure 1E). Based on these results, we found that LRGs in LUAD not only exhibited changes in mutation burden and copy number variation but also were associated with clinical prognosis in LUAD, highlighting the potential roles of LRGs in LUAD.

### Consensus clustering analysis of LRG molecular subtypes in LUAD

**Figure 2:**
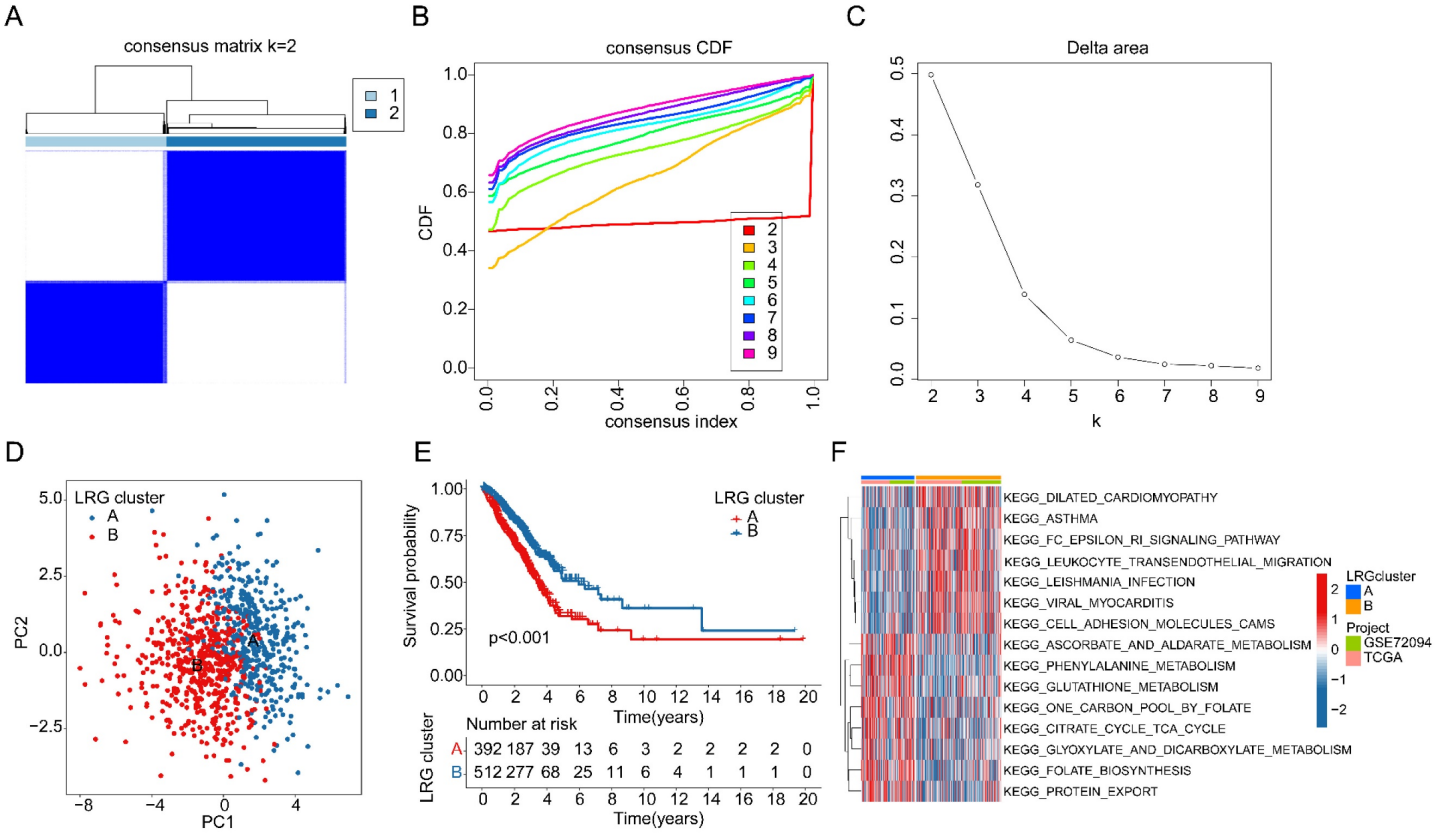
** Identification of LRG molecular subtypes in LUAD. (A-C)** Unsupervised consensus clustering to determine the optimal model parameters and clustering ratio for molecular subtypes. **(D)** PCA analysis of LRG molecular subtypes. **(E)** Evaluation of clinical prognosis outcomes for LRG molecular subtypes. **(F)** Predictive analysis of KEGG pathways for LRG molecular subtypes.

We extracted and included a total of 904 LUAD samples from the TCGA and GSE72094 databases to explore the molecular subtype characteristics of LRGs. Based on the optimal model parameters and classification ratio obtained from consensus clustering analysis, we categorized LUAD into two significantly distinct molecular subtype patterns. Specifically, LRG subtype A comprised 392 LUAD samples, while LRG subtype B comprised 512 LUAD samples (Figure 2A-C). The unsupervised PCA model results suggested a significant distinction between the two LRG molecular subtypes, indicating significant independence between them (Figure 2D). Clinical survival outcome analysis of LRG molecular subtypes indicated a significantly better clinical prognosis for LRG subtype B compared to LRG subtype A, suggesting that LUAD samples in LRG subtype B may exhibit better clinical survival benefits (Figure 2E). GSVA assessment results indicated significant upregulation of metabolism-related signaling pathways in LRG subtype A, including Phenylalanine Metabolism, Glutathione metabolism, Citrate cycle TCA cycle, and Glyoxylate and dicarboxylate metabolism, whereas immune-related signaling pathways were significantly upregulated in LRG subtype B, such as Leukocyte transendothelial migration and Cell Adhesion Molecules (CAMs) (Figure 2F). These results suggest that utilizing LRG gene signatures can accurately classify LUAD samples into two significantly different molecular subtype groups, which are correlated with the clinical survival prognosis of LUAD.

### Assessment of immune microenvironment infiltration features in LRG molecular subtypes

**Figure 3:**
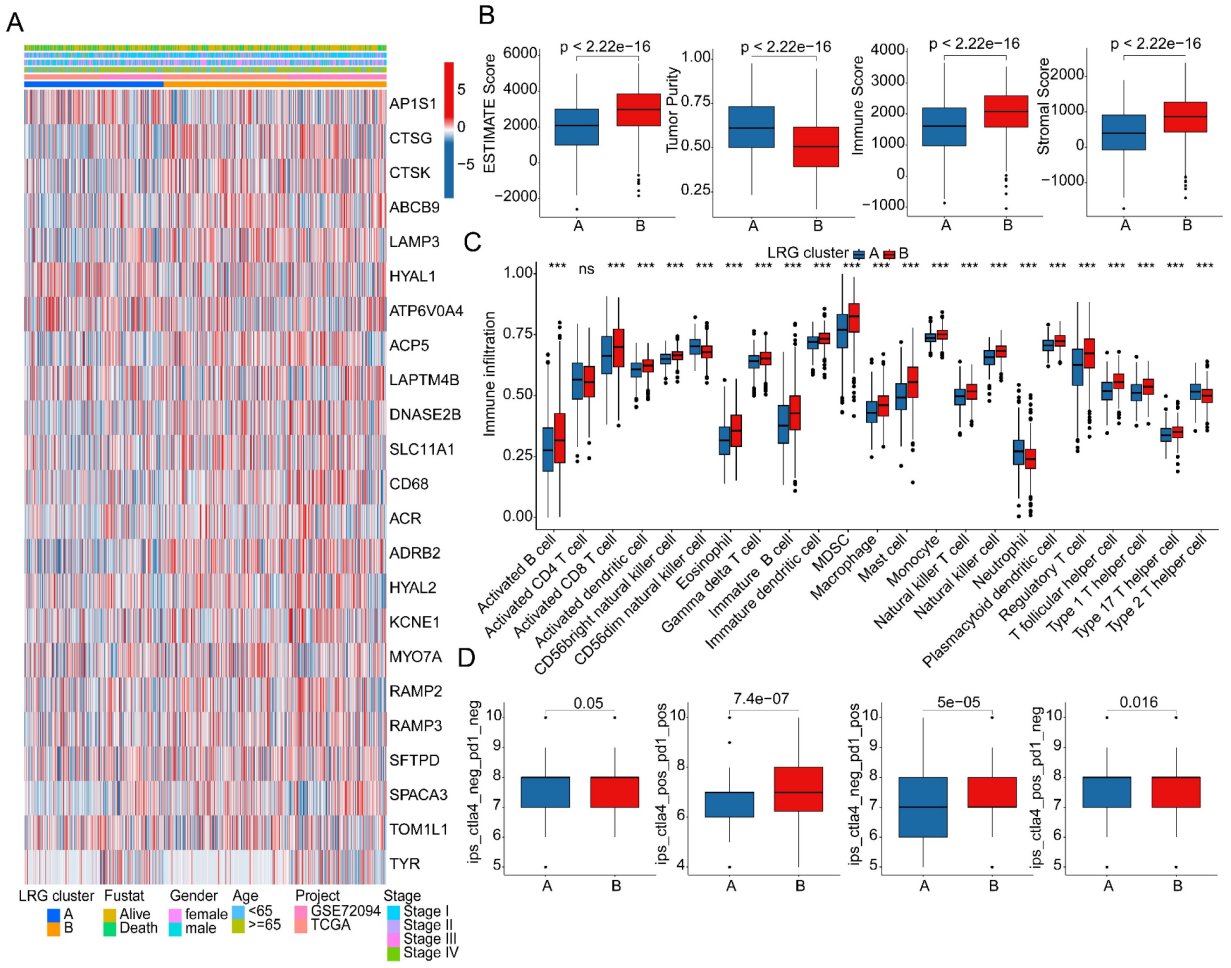
** Analysis of immune infiltration features and prediction of immune therapy response in LRG molecular subtypes. (A)** Expression profile analysis of LRG gene signatures in LRG molecular subtypes. **(B)** Evaluation of immune infiltration status in LRG molecular subtypes based on ESTIMATE algorithm. **(C)** Quantitative calculation of immune infiltrating cells in LRG molecular subtypes. **(D)** Evaluation of CTLA-4/PD-1 therapy response in LRG molecular subtypes.

As a prominent characteristic of tumors, the tumor immune microenvironment is closely associated with tumor invasion and treatment outcomes. In subsequent studies, we further elucidated the composition of the immune microenvironment in LUAD within LRG molecular subtypes. A heatmap displayed the expression patterns of differential LRG gene signatures in different LRG molecular subtypes (Figure 3A). Quantitative analysis of the immune infiltration status in LRG molecular subtypes was conducted using the ESTIMATE algorithm. The results indicated that in LRG subtype B, LUAD exhibited significantly higher ESTIMATE scores, stromal scores, and immune scores compared to LRG subtype A, while tumor purity was significantly lower in LRG subtype B. This suggests a significant reduction in immune infiltration status in LRG subtype B (Figure 3B). Based on the ssGSEA algorithm, we further elucidated the relative proportions of 23 immune infiltrating cells in LUAD within LRG subtypes. The results showed that in LRG subtype B, the proportions of most immune infiltrating cells were significantly increased, including activated B cells, CD8+ T cells, MDSCs, macrophages, etc. This indicates that a higher immune infiltration status may contribute to clinical survival benefits in LUAD samples (Figure 3C). Furthermore, based on the heterogeneity of immune infiltration characteristics in LRG molecular subtypes, we assessed the response of LRG molecular subtypes to immune therapy outcomes. IPS results suggested that LUAD samples in LRG subtype B exhibited a higher positivity rate when receiving immune therapy with CTLA-4 or PD-1, indicating better clinical benefits in LRG subtype B when undergoing CTLA-4/PD-1 treatment (Figure 3D). Based on these results, we found that the immune microenvironment infiltration characteristics in LUAD within LRG molecular subtypes are significantly different, and a higher immune status may contribute to clinical survival outcomes and immune therapy benefits in LUAD.

### Identification of differentially expressed genes in LRG molecular subtypes and identification of gene subtypes associated with LRG subtypes

**Figure 4:**
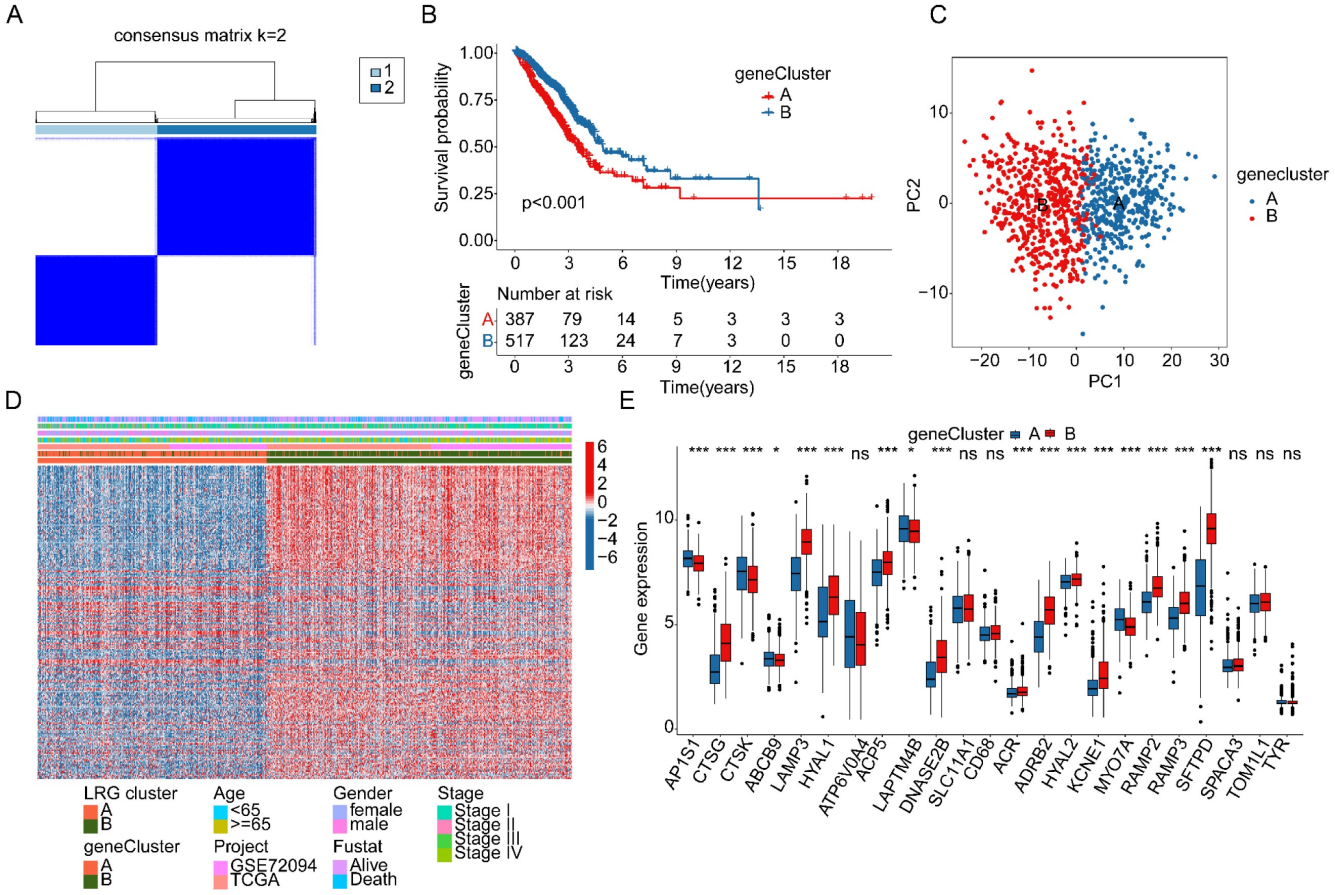
** Identification of gene subtypes associated with LRG molecular subtypes based on differentially expressed genes. (A)** Analysis of gene subtypes based on differentially expressed genes in LRG molecular subtypes. **(B)** Analysis of clinical prognosis outcomes in gene subtypes. **(C)** PCA model analysis of gene subtypes. **(D)** Distribution of differentially expressed genes in LRG molecular subtypes and different clinical pathological features. **(E)** Differential analysis of LRG gene signatures in gene subtypes.

In order to better understand the potential molecular mechanisms underlying LRG molecular subtypes, we utilized the "limma" script to explore differentially expressed genes (DEGs) between LRG molecular subtypes (Supplementary Figure 1A, |FC| > 2, p.adjust < 0.05). The results of GO enrichment analysis suggested that DEGs between LRG molecular subtypes were mainly involved in molecular functions such as leukocyte mediated immunity, humoral immune response, endocytic vesicle, and clathrin-coated vesicle (Supplementary Figure 1B). KEGG pathway enrichment analysis results indicated that phagosome, complement and coagulation cascades, and hematopoietic cell lineage may be important regulatory pathways between LRG molecular subtypes (Supplementary Figure 1C). Based on the DEGs between LRG molecular subtypes, we conducted secondary clustering analysis to classify LUAD samples into different gene subtypes related to LRG subtypes. According to the optimal partition ratio obtained from consensus clustering analysis (k=2), LUAD samples were randomly classified into two gene subtypes related to LRG subtypes, with 387 samples in gene subtype A and 517 samples in gene subtype B (Figure 4A). Clinical survival outcome analysis results showed that the survival probability of LUAD samples in gene subtype B was significantly higher than that in gene subtype A (p < 0.001, Figure 4B). Additionally, the PCA model plot revealed significant independence between molecular subtypes based on differential gene expression profiles (Figure 4C). Quantitative results indicated that in the gene molecular subtype group, the majority of DEGs were significantly upregulated in gene subtype B (Figure 4D). Furthermore, analysis of the expression profiles of LRG gene signatures revealed significant downregulation of AP1S1, CTSK, ABCB9, LAPTM4B, and MYO7A, while CTSG, LAMP3, HYAL1, ACP5, DNASE2B, ACR, ADRB2, HYAL2, KCNE1, RAMP2, RAMP3, and SFTPD were significantly upregulated in gene subtype B (Figure 4E).

### Development and independence assessment of LRG scoring system based on LRG subtype-associated DEGs

**Figure 5:**
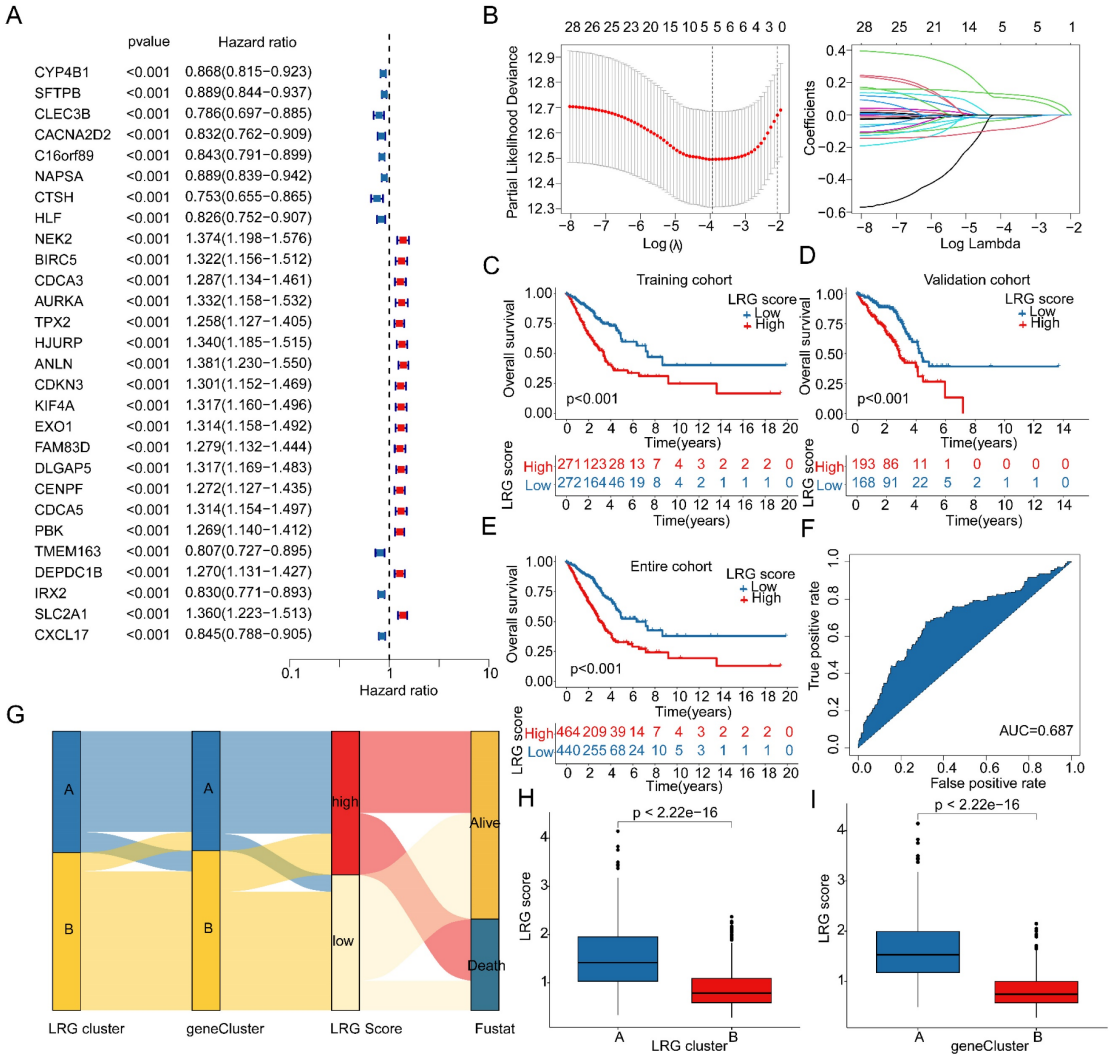
** Comprehensive analysis of LRG score prediction for clinical survival prognosis of LUAD. (A)** Univariate Cox analysis based on DEGs associated with LRG molecular subtypes. **(B)** Identification of feature gene signatures associated with LRG molecular subtypes using LASSO analysis. **(C-E)** Clinical survival probability analysis of LRG score subgroups in the training cohort, validation cohort, and complete cohort. **(F)** ROC curve analysis of LRG score prediction for LUAD survival prognosis. **(G)** Sankey diagram analysis of the relationships between LRG molecular subtypes, gene subtypes, LRG scoring system, and survival prognosis. **(H, I)** Differential analysis of LRG scores in LRG molecular subtypes and gene subtypes.

Based on the expression profiles of LRG molecular subtype-associated differential genes and clinical survival information, we developed a novel LRG scoring system to classify LUAD samples into different LRG score subgroups and evaluate their clinical prognosis. Using univariate Cox analysis, we identified 28 LRG variables associated with LUAD clinical prognosis from differential genes related to LRG molecular subtypes, including 17 risk variables and 11 protective variables (Figure 5A). Further feature variable selection was performed using LASSO regression analysis, identifying 5 feature variables based on optimal model parameters (Figure 5B). Subsequently, multivariate Cox analysis was employed to analyze prognostic variables with independent prognostic value and calculate the LRG score for each LUAD sample. With a 6:4 ratio, LUAD samples were randomly divided into training and validation cohorts, and based on the optimal cutoff for clinical survival, samples were stratified into low- and high-LRG score subgroups. Clinical survival outcome analysis revealed that in both the training and validation cohorts, the clinical survival outcome of the low-LRG score subgroup was significantly better than that of the high-LRG score subgroup, suggesting that LUAD samples with lower LRG scores may be associated with better prognostic outcomes (Figure 5C, D). In the entire cohort, the survival outcome of the low-LRG score subgroup was also significantly better than that of the high-LRG score subgroup (Figure 5E). Additionally, ROC curve analysis showed that the AUC for evaluating clinical prognosis of LUAD using LRG scores was 0.687, indicating a higher model accuracy (Figure 5F). Thus, based on the survival outcome analysis of LRG score subgroups in these three independent cohorts, we conclude that the LRG scoring system constructed based on differential genes associated with LRG molecular subtypes can accurately evaluate the clinical survival prognosis of LUAD samples, with higher LRG scores reflecting poor prognosis.

Further interpretation of the potential associations between LRG molecular subtypes, gene subtypes, LRG score subgroups, and LUAD survival prognosis was provided using a Sankey diagram (Figure 5G). Importantly, in both LRG molecular subtype and gene subtype subgroups, the LRG scores in subtypes with poor clinical prognosis (subtype A) were significantly higher than those in subtypes with good clinical prognosis (subtype B), further emphasizing the potential relationship between LRG scores and LUAD clinical survival prognosis (Figure 5H, I). Based on these results, we conclude that the LRG scoring system constructed based on LRG subtype-associated gene signatures can be independently used to evaluate the clinical survival prognosis of LUAD, while emphasizing the potential relationship between the LRG scoring system and LRG molecular subtypes.

### Correlation analysis between LRG score and clinical pathological features

**Figure 6:**
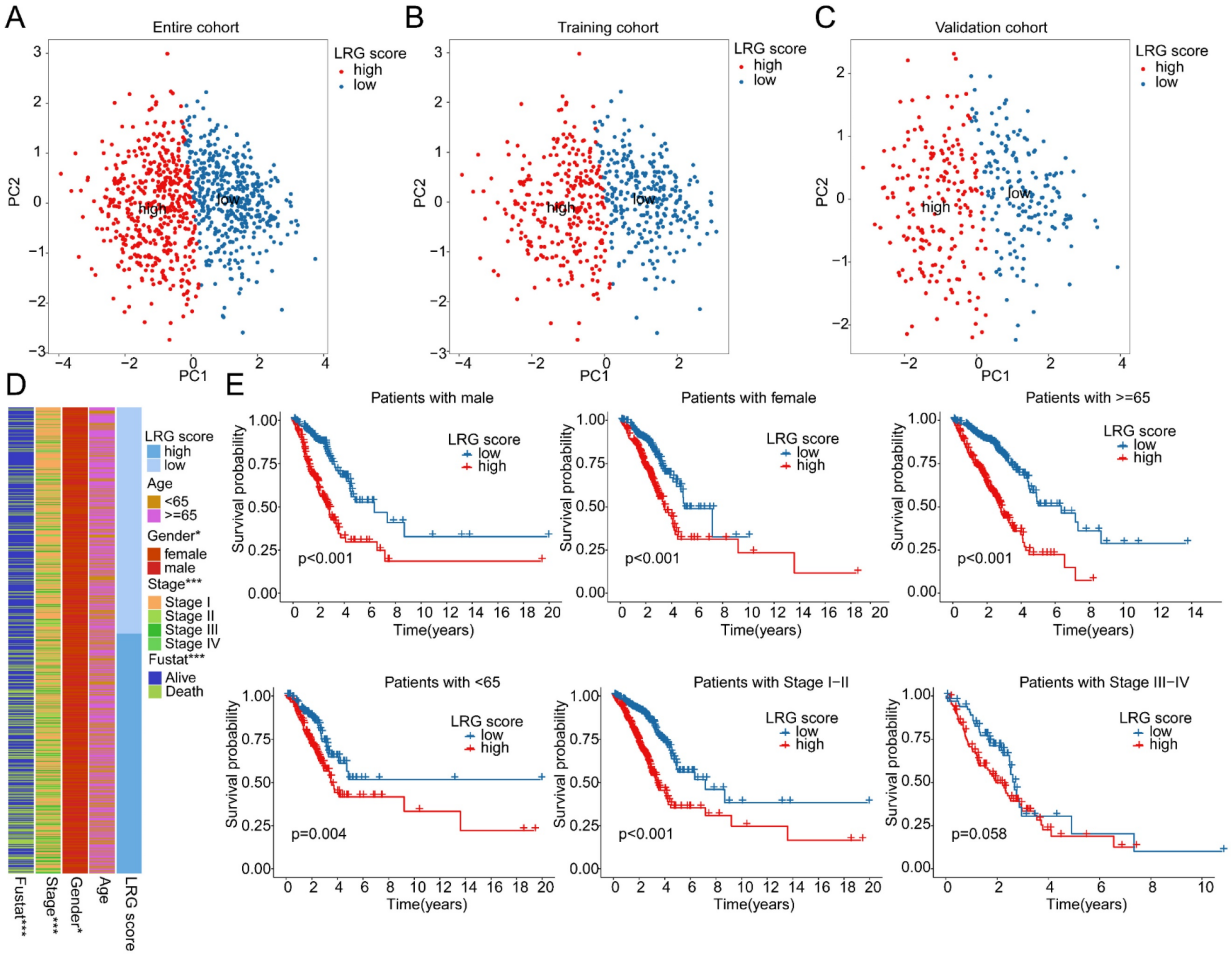
** Clinical prognosis survival analysis of LRG score subgroups in LUAD clinical pathological feature subgroups. (A-C)** PCA plot analysis based on the LRG scoring system in the complete cohort, training cohort, and validation cohort. **(D)** Distribution of LRG scores in different clinical pathological features of LUAD samples. **(E)** Clinical prognosis analysis of LUAD samples based on clinical pathological feature subgroups using the LRG scoring system.

The PCA plot results indicated that in the entire cohort, training cohort, and validation cohort, the LRG score system could accurately distinguish between LRG score subgroups (Figure 6A-C). Therefore, we further explored the relationship between LRG scores and different clinical-pathological features of LUAD. The results of differential expression analysis suggested significant differences in LRG scores among different clinicopathologic features (Supplementary Figure 2A-C). Moreover, Significant differences were observed in the distribution of LRG scores among clinical-pathological features such as gender, stage, and survival status of LUAD samples (Figure 6D). The correlation analysis revealed a significant association between the LRG signature and the clinicopathological characteristics of LUAD. Specifically, gender and stage were significantly positively correlated with the LRG scoring system, whereas age was significantly negatively correlated, highlighting the potential prognostic value of the LRG signature in LUAD (Supplementary Figure 2D, E). Differential expression analysis further indicated that the expression of the LRG signature varied markedly across different clinicopathological features of LUAD (Supplementary Figure 2F). Clinical survival curve results suggested that in subgroups based on gender, stage, and age (<65 and ≥65), LUAD samples with low LRG scores exhibited significantly better survival probabilities than those with high LRG scores. Notably, in the stage III-IV subgroup, there was no significant difference in clinical survival outcomes between LRG score subgroups, possibly due to the smaller sample size in this subgroup (Figure 6E). Based on these results, we speculate that the use of LRG scores can predict the clinical prognosis of LUAD samples across different clinical-pathological features.

### Integrated analysis of the independent prognostic value of LRG score and clinical pathological features

**Figure 7:**
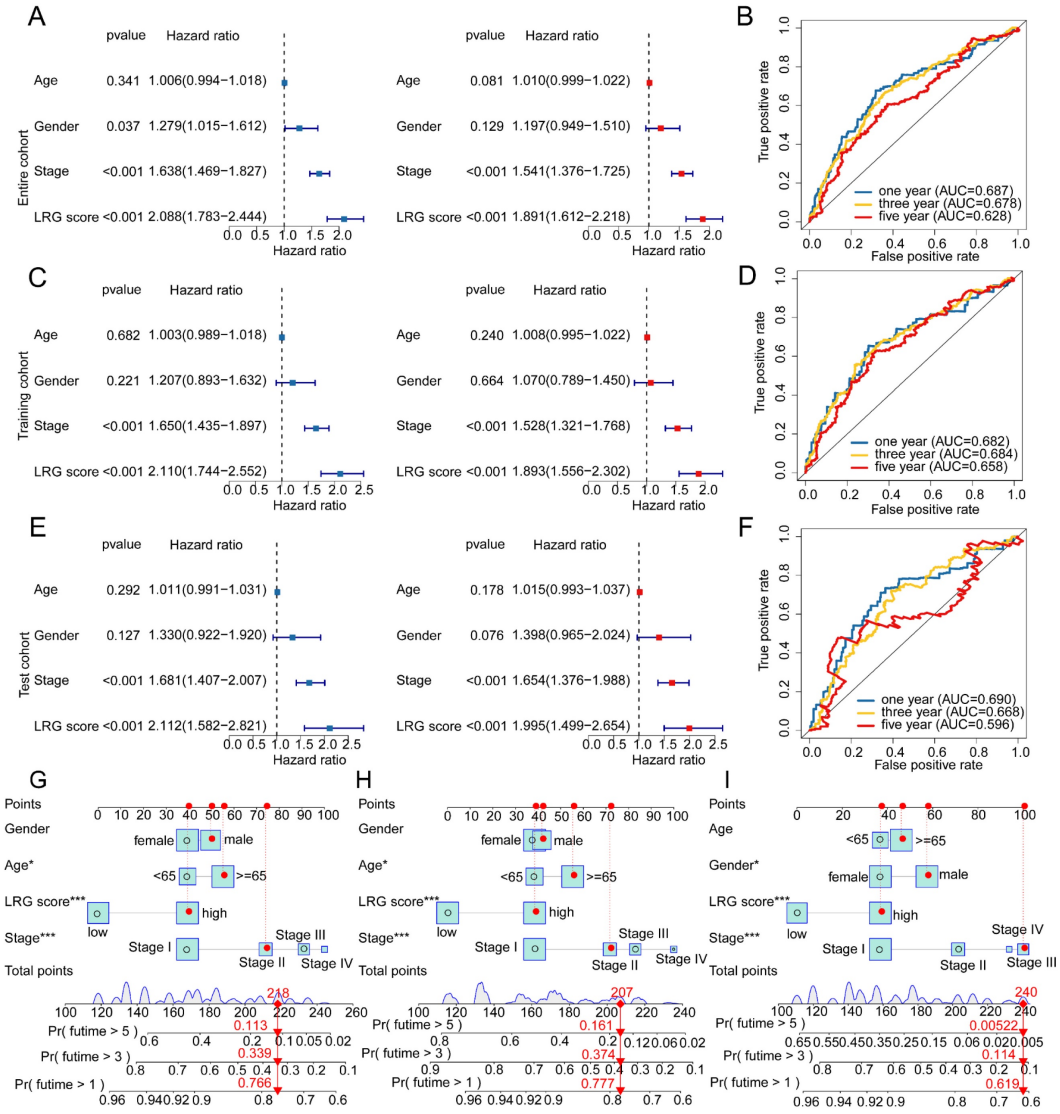
** Independent prognostic value prediction of the LRG scoring system and construction of the nomogram model. (A)** Univariate and multivariate COX analysis based on the LRG scoring system and clinical pathological variables in the complete cohort. **(B)** Time-dependent curve analysis for different time points in the complete cohort. **(C-F)** Univariate and multivariate COX analysis based on LRG scoring and clinical pathological feature variables, as well as time-dependent curve evaluation, in the training cohort and validation cohort. **(G-I)** Nomogram model constructed based on LRG scoring system and clinical pathological feature variables in each independent cohort.

Given the predictive value of LRG scores in LUAD clinical-pathological feature subgroups, we integrated the LRG scoring system with clinical-pathological feature variables to further elucidate the independent prognostic value of each variable in predicting LUAD clinical survival prognosis. In the entire cohort, both univariate and multivariate Cox analysis results of clinical-pathological feature variables and LRG scores showed that stage and LRG scores were associated with poor prognosis in LUAD, with stage and LRG scores serving as risk factors (Figure 7A). Additionally, in the entire cohort, the time-dependent ROC curve AUC values for 1-, 3-, and 5-year survival were 0.687, 0.678, and 0.628, respectively (Figure 7B). In the training cohort and validation set, univariate and multivariate Cox analysis results based on LRG scores and clinical-pathological feature variables showed that stage and LRG scores were associated with poor prognosis in LUAD, with LRG scores having a more significant HR than stage, consistent with the risk analysis results of the entire cohort. Furthermore, in the training cohort, the AUC values of the ROC curve for 1-, 3-, and 5-year survival were 0.682, 0.684, and 0.658, respectively, while in the validation set, the AUC values were 0.690, 0.668, and 0.596, respectively (Figure 7C-F). These results suggest that LRG scores may serve as an independent prognostic indicator for LUAD and can accurately predict its clinical survival outcomes. To better illustrate the predictive ability of the LRG scoring system for 1-, 3-, and 5-year survival probabilities of LUAD samples, we constructed a new nomogram model based on LRG scores and clinical-pathological feature variables and calculated the survival probabilities for different time points (Figure 7G-I). Based on the above results, we conclude that the LRG scoring system is an independent indicator for predicting the clinical survival prognosis of LUAD samples, and the nomogram model constructed based on LRG scores and clinical-pathological feature variables can accurately predict the survival probabilities of LUAD samples at different time points.

### Analysis of immune microenvironment infiltration characteristics of LRG score subgroups

**Figure 8:**
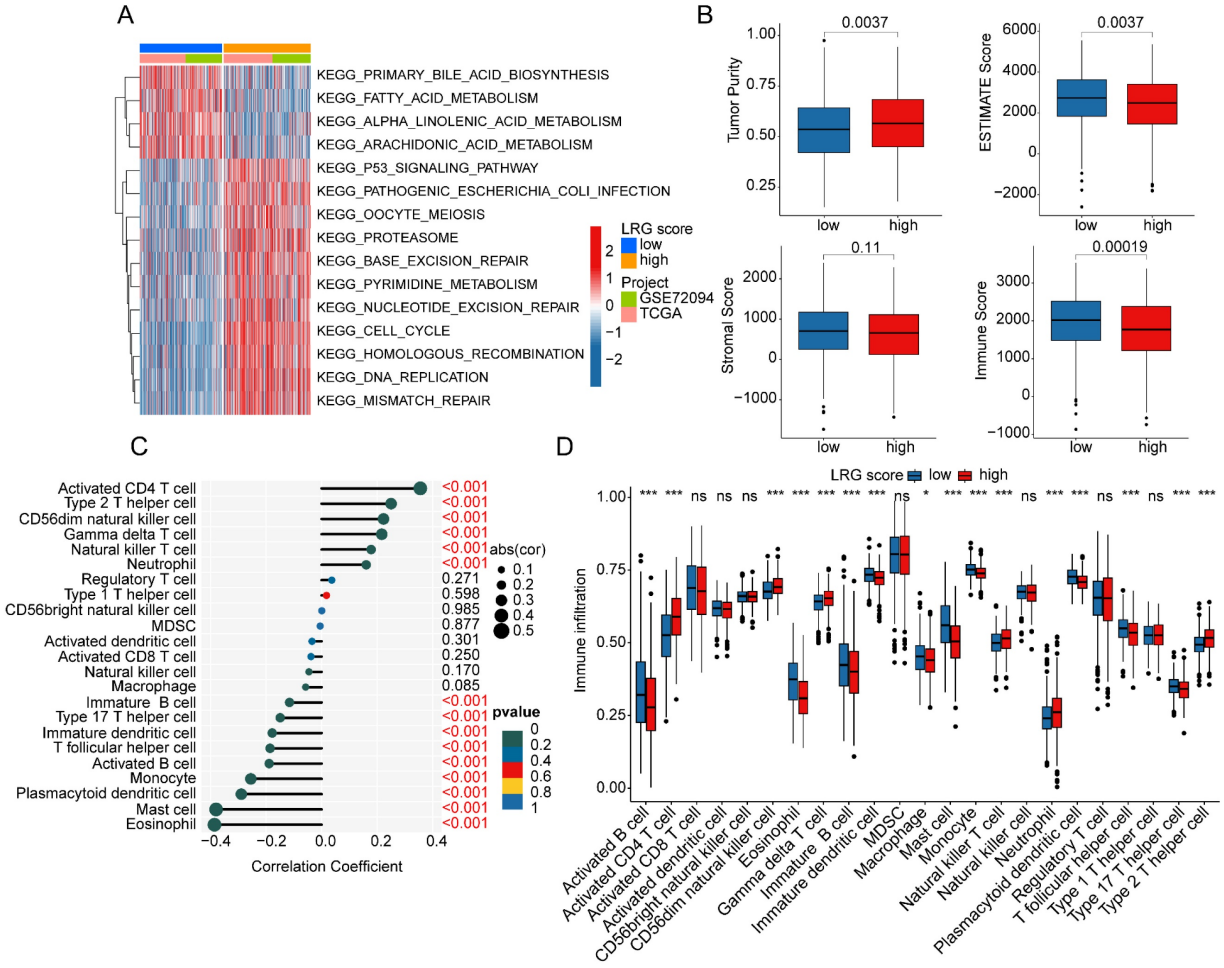
** Analysis of immune infiltration characteristics and molecular regulatory mechanisms in LRG score subgroups. (A)** Differential analysis of KEGG signaling pathways in LRG score subgroups. **(B)** Prediction of immune infiltration status in LRG score subgroups. **(C)** Correlation analysis between LRG score and immune microenvironment. **(D)** Evaluation of immune infiltration characteristics in LRG score subgroups.

To elucidate the regulatory mechanisms underlying the prognostic differences in LUAD samples among LRG score subgroups, we utilized GSVA analysis to evaluate the KEGG signaling pathways and immune microenvironment infiltration characteristics between LRG score subgroups. The results of molecular regulatory mechanisms indicated that in the low LRG score subgroup, pathways such as primary bile acid biosynthesis, fatty acid metabolism, alpha-linolenic acid metabolism, and arachidonic acid metabolism were significantly upregulated. Conversely, in the high LRG score subgroup with poorer prognosis, tumor-related signaling pathways and nucleotide-related signaling pathways were significantly upregulated, including the p53 signaling pathway, cell cycle, nucleotide excision repair, and DNA replication (Figure 8A). Additionally, we observed significant changes in the immune status among LRG score subgroups. In the high LRG score subgroup with poorer prognosis, tumor purity was significantly higher than that in the low LRG score subgroup, while ESTIMATE score and immune score were significantly lower than those in LRG score subgroup A (Figure 8B). Using the ssGSEA algorithm, we further quantified the relative proportions of 23 immune cells in LRG score subgroups. The correlation analysis between LRG score and immune infiltration features indicated that LRG score was significantly positively correlated with neutrophil, natural killer T cell, gamma delta T cell, CD56dim natural killer cell, type 2 T helper cell, and activated CD4 T cell, while it was significantly negatively correlated with eosinophil, mast cell, plasmacytoid dendritic cell, monocyte, activated B cell, T follicular helper cell, immature dendritic cell, type 17 T helper cell, and immature B cell (Figure 8C). The quantitative analysis of immune cells showed significant differences in the proportions of most immune cells in the high LRG score subgroup, such as activated B cell, activated CD4 T cell, CD56dim natural killer cell, and eosinophil (Figure 8D). These results suggest that the immune infiltration status of LUAD varies significantly among LRG score subgroups, which may be an important molecular mechanism underlying the differences in clinical survival prognosis.

### Analysis of mutation landscape features and immune therapy response assessment in LRG score subgroups

**Figure 9:**
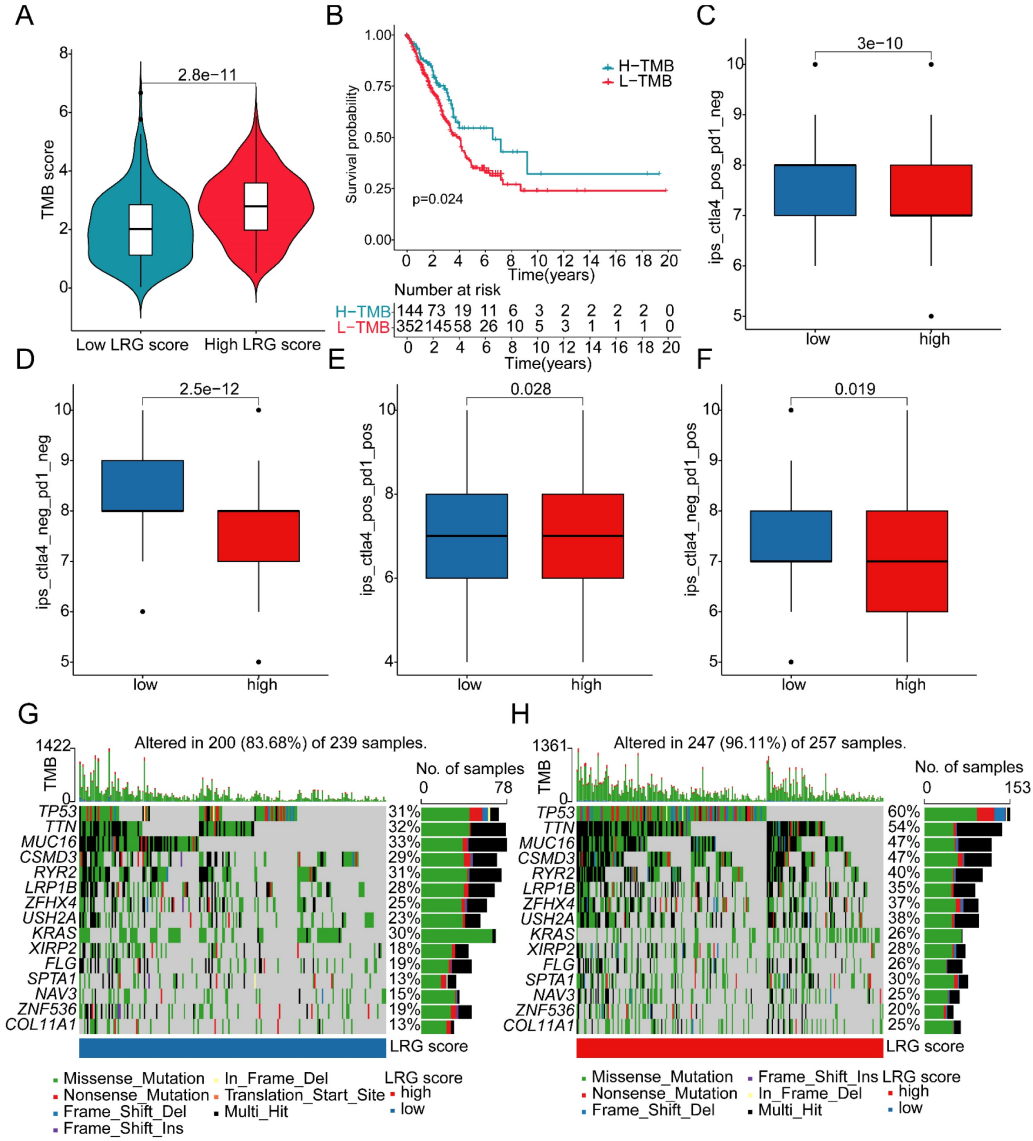
** Somatic mutation characteristics and immune therapy response assessment in LRG score subgroups. (A)** Differential analysis of TMB scores in LRG score subgroups. **(B)** Clinical survival outcome analysis of high and low TMB subgroups. **(C-F)** Prediction of immune therapy response levels in LRG score subgroups. **(G, H)** Analysis of somatic mutation characteristics in LRG score subgroups.

**Figure 10:**
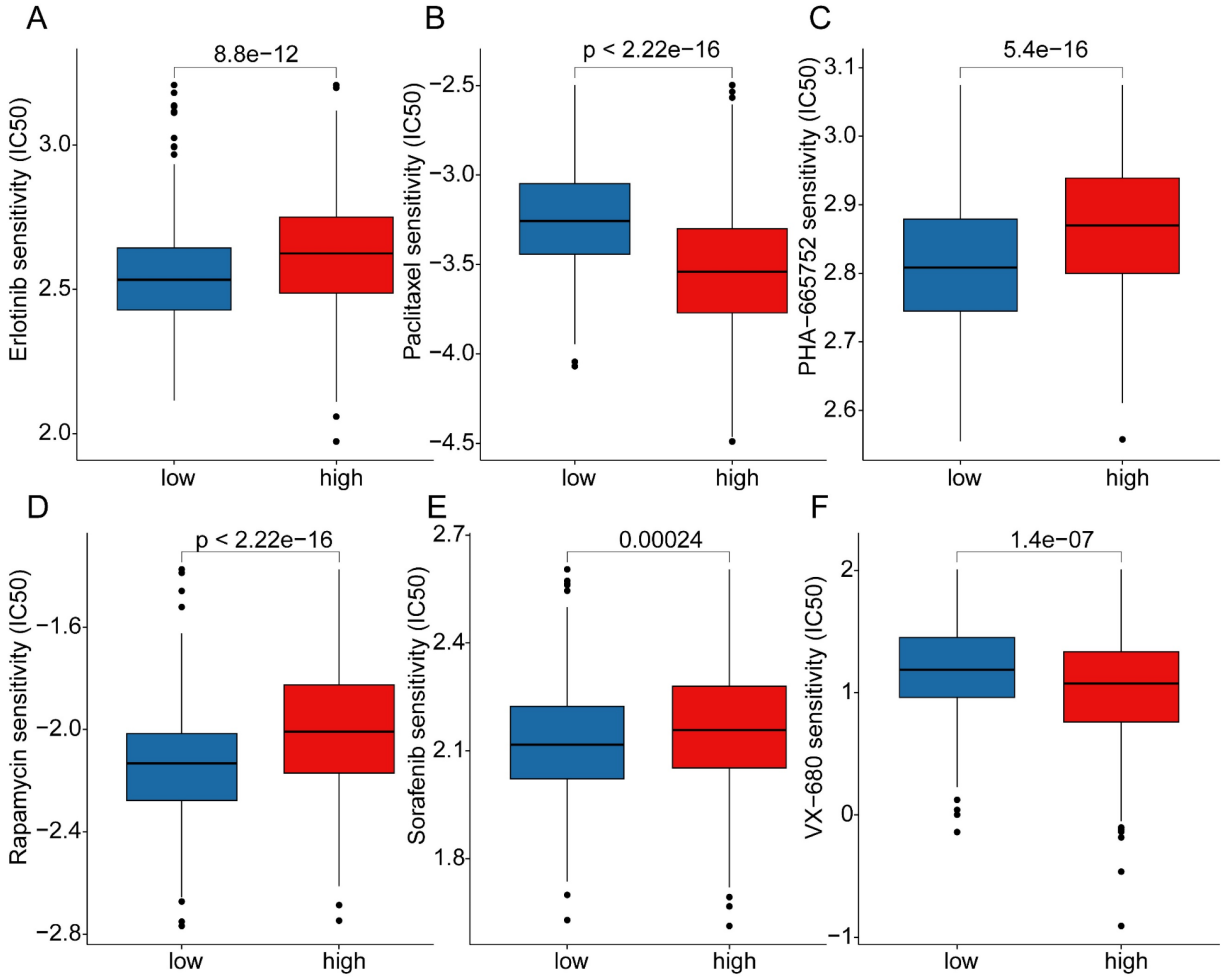
** Drug sensitivity analysis in LRG score subgroups. (A)** Erlotinib, **(B)** Paclitaxel, **(C)** PHA-665752, **(D)** Rapamycin, **(E)** Sorafenib, **(F)** VX-680 differential analysis in LRG score subgroups.

Somatic mutation burden, as a crucial indicator of tumors, has been reported to be closely associated with immune therapy. In the subsequent study, we further elucidated the mutation burden characteristics and the response to immune therapy in LRG score subgroups. The results of mutation burden characteristics indicated that in the high LRG score subgroup, the TMB score was significantly higher than that in the low LRG score subgroup, suggesting that LUAD samples in the high LRG score subgroup may exhibit higher mutation burden characteristics (Figure 9A). Furthermore, we observed that LUAD samples with higher mutation burden had better clinical survival prognosis (Figure 9B). Based on the variation characteristics of mutation burden, we predicted the response of LRG score subgroups to CTLA-4 and PD-1 immune therapy. The results showed that in the low LRG score subgroup, the IPS score of LUAD was significantly higher than that in the high LRG score subgroup, implying that the population in the high LRG subgroup may have more clinical treatment benefits from CTLA-4 and PD-1 immune therapy (Figure 9C-F). We further demonstrated the significantly mutated somatic mutation gene signature in LRG score subgroups. In the high LRG score subgroup, most gene signatures exhibited significantly higher mutation characteristics, such as TP53 (high vs low: 60% vs 31%), TTN (high vs low: 54% vs 32%), MUC16 (high vs low: 47% vs 33%), CSMD3 (high vs low: 47% vs 29%), and RYR2 (high vs low: 40% vs 31%) (Figure 9G, H). Additionally, we predicted potential beneficial small molecule compounds in LRG score subgroups based on the GDSC database. As shown in **Figure 10A-F**, drug sensitivity results suggested that LUAD samples in the low LRG score subgroup might have a better response to VX-680 and Paclitaxel, while LUAD samples in the high LRG score subgroup might have a better response to Erlotinib, PHA-665752, Rapamycin, and Sorafenib. These results elucidate that the high LRG score subgroup exhibits higher mutation burden characteristics and reflects the response to immune therapy and drug therapy, providing new insights and perspectives for the precision treatment of LUAD.

### Single-cell sequencing analysis reveals the composition of cell subpopulations and the expression characteristics of prognostic signatures in LUAD

**Figure 11:**
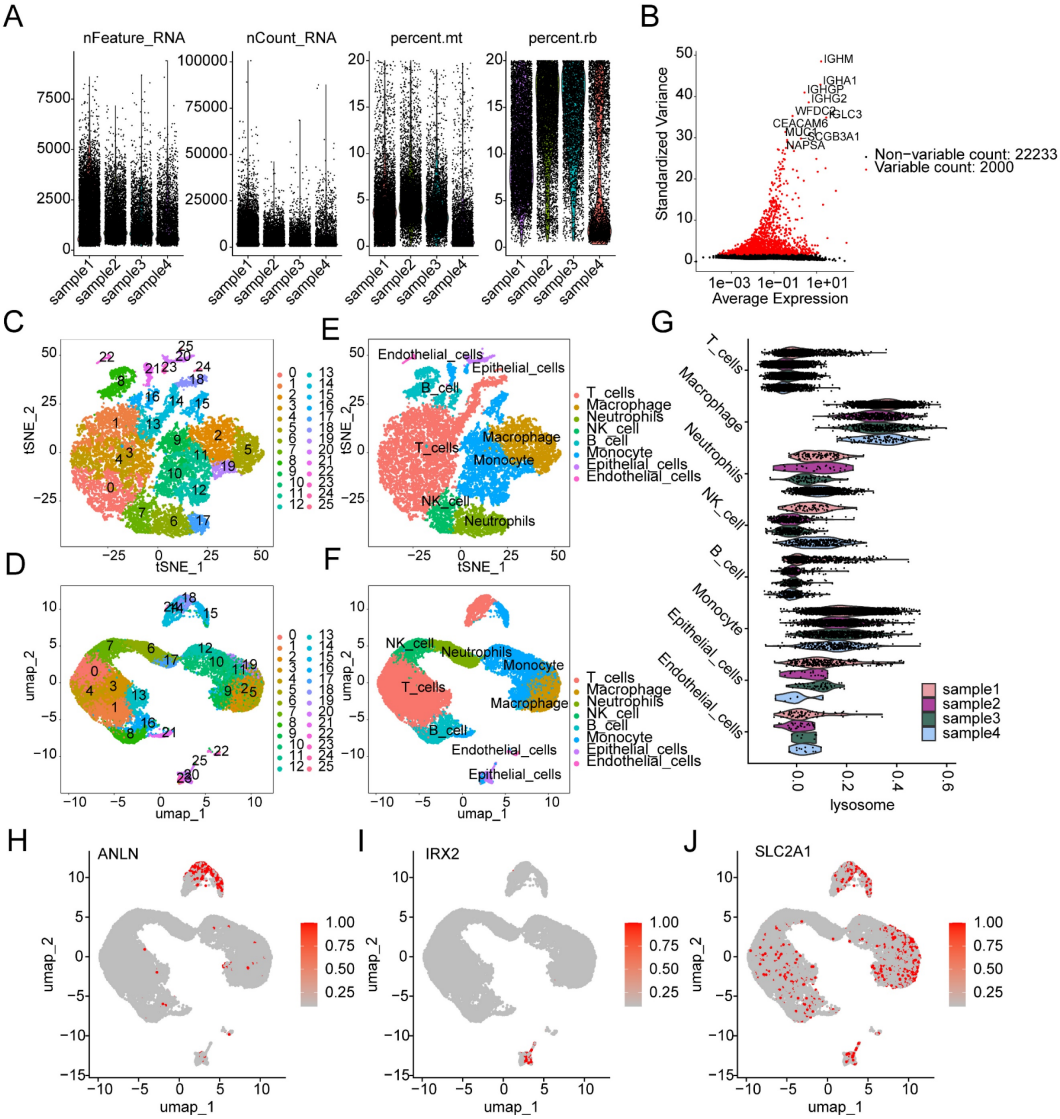
** Single-cell sequencing analysis reveals the classification of cell subpopulations and the expression characteristics of prognostic signatures in LUAD. (A)** Quality control and normalization of single-cell sequencing data. **(B)** Identification of the top 2000 highly variable genes. **(C, D)** t-SNE and UMAP dimensionality reduction plots showing the classification of 25 cell subpopulations. **(E, F)** t-SNE and UMAP dimensionality reduction plots for cell type analysis. **(G)** Expression distribution of the LRG signature across different cell types. **(H-J)** UMAP plots showing the expression characteristics of the LRG molecular subtype prognostic signature across different cell types.

We further elucidated the composition of cell subpopulations in LUAD and the distribution landscape of LRG subtype-associated prognostic signatures at the single-cell sequencing level. Using the GSE223923 dataset, we extracted single-cell sequencing data from four LUAD samples for subsequent analysis. After performing quality control and normalization for each sample, we identified 2000 highly variable genes for further dimensionality reduction analysis (Figure 11A, B). Based on marker genes from the CellMarker database, we accurately identified 25 cell subpopulations in LUAD (Supplementary Figure 3A). t-SNE and UMAP dimensionality reduction plots revealed the distribution characteristics of these 25 cell subpopulations (Figure 11C, D). Cell type scoring and annotation using the singleR algorithm identified eight distinct annotated subpopulations: T cells, Macrophages, Neutrophils, NK cells, B cells, Monocytes, Epithelial cells, and Endothelial cells (Supplementary Figure 3B). t-SNE and UMAP plots further illustrated the distribution patterns of these annotated cell types (Figure 11E, F). Violin plots indicated that the LRG signature showed significant expression in various cell types, including T cells, Monocytes, and Macrophages (Figure 11G). Additionally, we explored the expression profiles of LRG subtype-associated prognostic signatures in different cell types. UMAP analysis showed that ANLN was highly expressed in T cells and Monocytes, IRX2 was predominantly expressed in Epithelial cells, while SLC2A1 was expressed at high levels in Macrophages, T cells, Monocytes, B cells, and Epithelial cells (Figure 11H-J).

### SLC2A1 facilitates proliferation and migration of lung cancer cells

**Figure 12:**
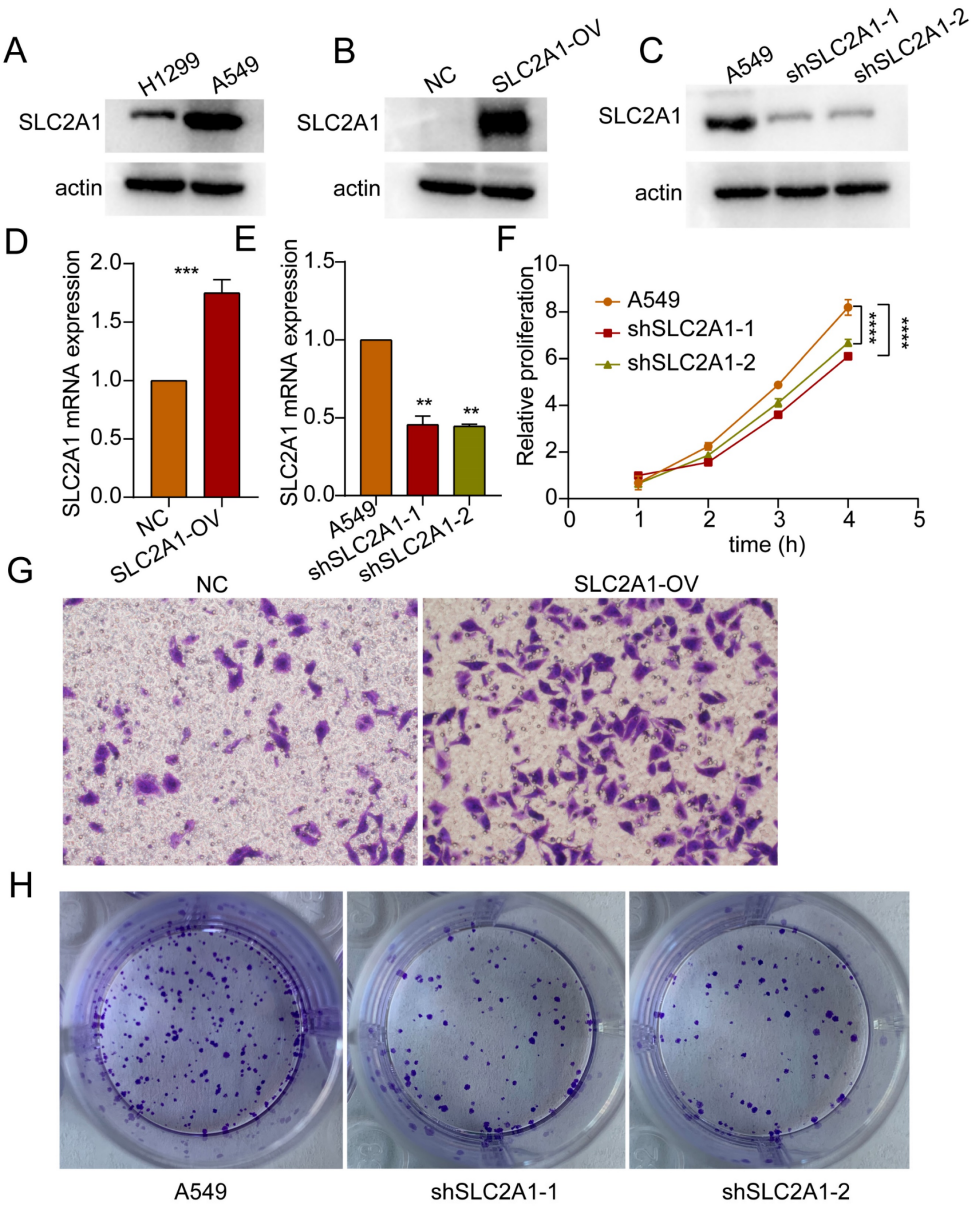
** SLC2A1 promotes proliferation and migration of lung cancer. (A)** Western blot experiments detected basal expression levels in H1299 and A549 cells. **(B, C)** Western blot validate the overexpression and knockdown efficiency of SLC2A1. **(D, E)** The mRNA expression of SLC2A1 in overexpression and knockdown cell lines. **(F)** Proliferation assay assessing the impact of SLC2A1 knockdown on proliferation capacity of A549 cells. **(G)** Transwell assay assessing the effect of SLC2A1 overexpression on migration ability of H1299 cells. **(H)** Colony formation assay assessing the impact of SLC2A1 knockdown on colony formation capacity of A549 cells. *p < 0.05, **p < 0.01, ***p < 0.001, ****p < 0.0001, student's t-test, n ≥ 3.

Based on the HR values of the selected markers, we selected SLC2A1 for further study in order to verify our bioinformatics results. Initially, the baseline expression levels of SLC2A1 protein were assessed in lung cancer H1299 and A549 cells, revealing relatively higher expression in A549 (Figure 12A). Subsequently, SLC2A1-overexpressing cell lines were established in H1299 with relatively lower expression levels, while SLC2A1 knockdown cell lines were established in A549 with relatively higher expression levels (Figure 12B, C). The mRNA results further validated the efficiency of SLC2A1 overexpression and knockdown in cell lines (Figure 11D, E). Proliferation assays indicated that knockdown of SLC2A1 inhibited the proliferation capacity of tumor cells (Figure 12F). Colony formation assays and Transwell assays revealed that high expression of SLC2A1 promoted tumor migration (Figure 12G, H).

## Discussion

Our findings support the significant role of lysosomes in LUAD. Although research in this area is still incomplete, previous reports have indicated the important role of lysosomes in the development of LUAD. Enhanced lysosomal function promotes ferroptosis in LUAD and enhances the efficacy of cisplatin in lung adenocarcinoma 20. Targeting specific proteins in LUAD cancer cells and inducing lysosomal degradation has the potential to inhibit tumor development 21. Additionally, autophagy has been reported to be associated with increased tumor invasiveness and epithelial-mesenchymal transition in LUAD 22, 23. Targeting autophagy can suppress chemoresistance in LUAD patients 24. We further demonstrated that effective risk stratification of LUAD patients can be achieved through LRGs and provided new possible intervention target.

Immunotherapy holds great potential in LUAD 25. However, many LUAD patients still experience limited benefits from immunotherapy 26. Further refinement of clinical and molecular characteristics that predict the effectiveness of immunotherapy in LUAD is crucial 27. Our results demonstrate significant differences in immunotherapy responsiveness based on LRGs and LRG-based subtyping, further refining molecular characteristics for stratifying immunotherapy efficacy. Through a comprehensive analysis of immune evasion mechanisms, the characteristics of immune cell infiltration in the tumor microenvironment and the mechanisms of immune checkpoint inhibition 28, 29, machine learning applied to tumor immunogenicity has been shown to facilitate the development of precision immunotherapy 30. Therefore, our subtyping approach holds potential clinical significance.

We found an association between higher TMB and poorer prognosis in the high LRG score group. TMB has been identified as a predictive factor for clinical response to immune checkpoint inhibitors (ICI) 31. A higher mutation burden often increases tumor neoantigen production, thereby activating immune responses; however, in some cases, this mutation burden may lead tumor cells to develop immune evasion mechanisms, such as the overexpression of immune checkpoint molecules, which suppress antitumor immunity 32, 33. Furthrmore, immunosuppressive cells in the tumor microenvironment are more likely to appear in tumors with a high mutation burden, further impacting prognosis 34. Our results also indicate the predictive significance of this signature for immunotherapy efficacy based on different TMB scores by risk stratification. Furthermore, reports suggest that MTSS1 reduces immune evasion in LUAD by promoting AIP4-mediated PD-L1 monoubiquitination and lysosomal degradation, highlighting the potential of lysosomal function as a predictor of immunotherapy effectiveness 35.

Our results show lower eosinophil levels in LUAD subgroups with poorer prognosis. LUAD can exhibit symptoms of increased eosinophils 36. Additionally, lung cancer cells can produce eosinophilopoietic factors, suggesting a potential correlation between lung cancer and eosinophils 37. However, the specific mechanisms underlying their relationship in LUAD are unclear. Similar reports in other tumor types indicate that peritumoral eosinophils can predict favorable outcomes 38. Evidence suggests that treatment with pembrolizumab for non-small cell lung cancer leads to increased eosinophil counts 39. Moreover, treatment processes for LUAD, including Osimertinib therapy and anti-PD-1-related treatments, can induce eosinophilic pneumonia 39-41. Therefore, differences in treatment regimens may contribute to variations in prognosis and eosinophil levels. However, we did not obtain information on treatment differences between different groups.

Our results demonstrate that SLC2A1, as a participant in autophagy and lysosome-related processes, promotes proliferation and migration of lung cancer. Autophagy maintains the metabolism of stressed cells by promoting intracellular degradation and nutrient recycling. Increased surface expression of SLC2A1 on cells can enhance glucose uptake and glycolytic flux to meet increased glycolytic demands 42. Mis-localization of SLC2A1 to lysosomes can affect glucose uptake, thereby activating the AMPK-ULK1 pathway, sensitizing cancer cells to energy stress, and inhibiting tumor growth 43. Differential expression of SLC2A1 exists in various tumors 44, 45. In LUAD, increased SLC2A1 expression after surgical resection of lung adenocarcinoma is associated with poor prognosis 46. Additionally, several long non-coding RNAs (lncRNAs) have been reported to promote proliferation and invasion of LUAD by inducing SLC2A1 expression 47, 48. Therefore, targeting SLC2A1 to increase cancer cell autophagic flux holds potential clinical significance for cancer treatment.

In summary, by applying a variety of advanced bioinformatic analysis, we developed an LRG prognostic model. This model provides a more accurate risk prediction for the clinical prognosis of LUAD from multiple perspectives, enhancing the precision of prognosis and offering valuable support for personalized treatment strategies. The study highlights the significance of immunotherapy strategies and lysosomal targeted therapy in LUAD. Additionally, the LRG scoring system and the nomogram model offers a practical tool for clinical application, while* in vitro* validation of SLC2A1 opens new directions for potential targeted therapies in LUAD. However, analyses based on public databases do have limitations. Biases introduced by an insufficient sample size may affect prediction accuracy, highlighting the need to increase sample size in future studies to further validate the reliability of the results. Data heterogeneity and batch effects are also unavoidable in existing database analyses. The limitations of public databases in terms of time and geographical scope underscore the importance of further multicenter and long-term studies. By incorporating the expression characteristics of LRGs for prognostic evaluation of LUAD patients, and further investigating the role of LRGs in different cell subpopulations through single-cell sequencing analysis, we provide a deeper understanding of their functional significance. Given substantial evidence that lysosomes are involved in the development and progression of LUAD, we believe that further research focusing on lysosomes and their associated genes has valuable academic significance and potential clinical implications.

## Supplementary Material

Supplementary figures and tables.

## Figures and Tables

**Table 1:** Baseline characterization of LUAD samples in GEO and TCGA database.

Dataset name	GSE72094 dataset	TCGA dataset
Sample type		
Normal	/	59
Lung Adenocarcinoma	397	507
Primary tumour, n (%)	397(100%)	507(100%)
Sex, n (%)		
Female	221(55.67%)	306(54.06%)
Male	176(44.33%)	260(45.94%)
Age, n (%)		
≥65	291(73.30%)	308(54.42%)
<65	106(26.70%)	248(53.82%)
others	/	10(1.77%)
Stage, n (%)		
Stage 0	/	/
Stage I	254(63.98%)	302(53.36%)
Stage II	66(16.62%)	133(23.50%)
Stage III	57(14.36%)	94(16.61%)
Stage IV	15(3.78%)	28(4.95%)
others	5(1.26%)	9(1.59%)
T stage, n (%)		
T 0	/	/
T I	/	188(33.22%)
T II	/	308(54.42%)
T III	/	47(8.30%)
T IV	/	20(3.53%)
others	/	3(0.53%)
M stage, n (%)	/	
M 0	/	378(66.78%)
M 1	/	27(4.77%)
M X	/	156(27.56%)
others	/	5(0.88%)
N stage, n (%)	/	
N 0	/	357(63.07%)
N 1	/	107(18.90%)
N 2	/	84(14.84%)
N 3	/	2(0.35%)
N X	/	15(2.65%)
others	/	1(0.18%)
