# Supplementary material for: Multi-omics analysis reveals lysosome-associated molecular subtype characterization and prognostic modeling system in lung adenocarcinoma
Source: J Cancer. 2025 Feb 18;16(6):1794–813. doi: 10.7150/jca.105351 (PMC11905399; doi:10.7150/jca.105351)

Supplementary figure 1. Differential gene expression analysis among LRG molecular subtypes. (A) Volcano plot analysis of differentially expressed genes among LRG molecular subtypes. Differential thresholds: |fold change| ≥ 2 and p.adjust < 0.05, with red indicating upregulated genes and blue indicating downregulated genes. (B, C) KEGG and GO enrichment analyses of differentially expressed genes associated with LRG molecular subtypes.

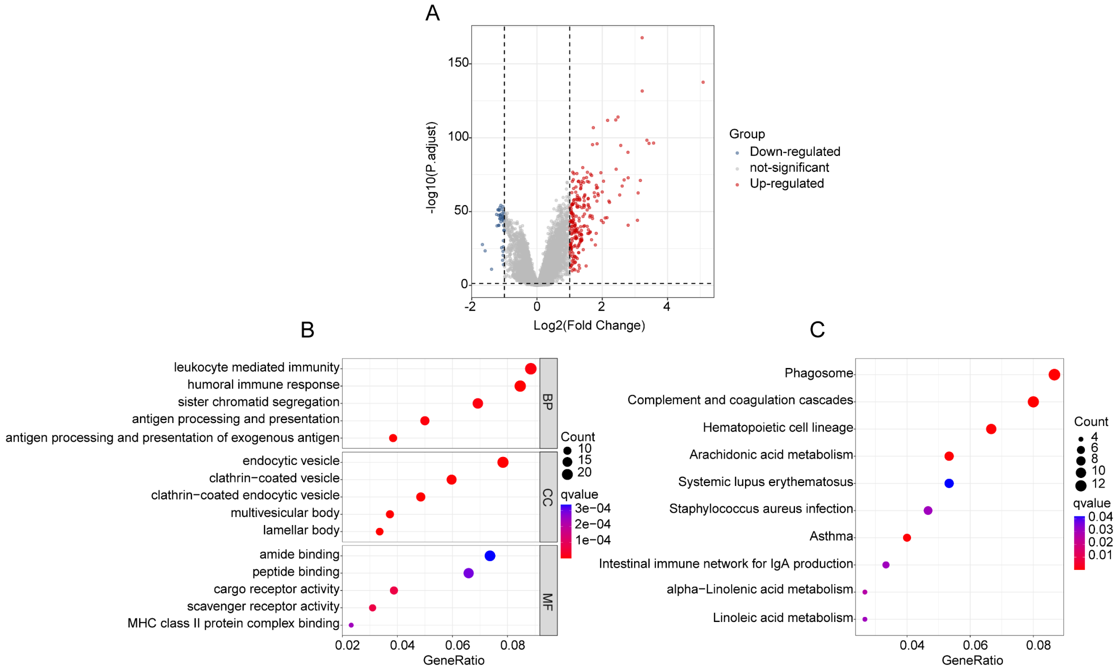

Supplementary figure 2. Analysis of the potential correlation between clinicopathological characteristics and the LRG scoring system in LUAD. (A-C) Differential expression analysis of LRG scores across clinicopathological subgroups. (D) Correlation analysis between LRG expression profiles and clinicopathological characteristics. (E) Correlation analysis between the LRG scoring system and clinicopathological characteristics. (F) Expression analysis of the LRG signature across different clinicopathological characteristics.

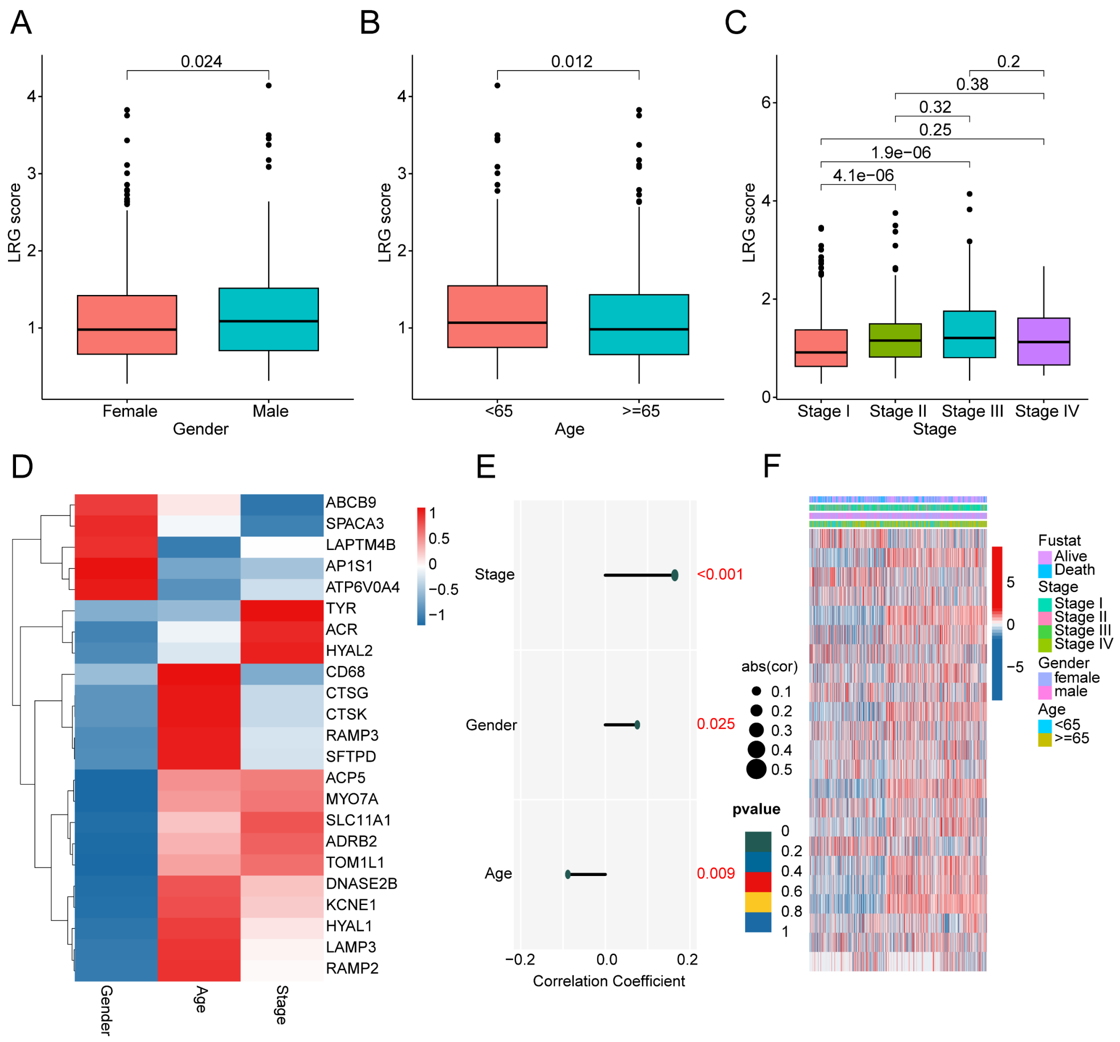

Supplementary figure 3. Cell type identification based on single-cell sequencing data. (A) Expression profiles of marker genes across different molecular subgroups. (B) Cell type identification using the SingleR annotation algorithm.

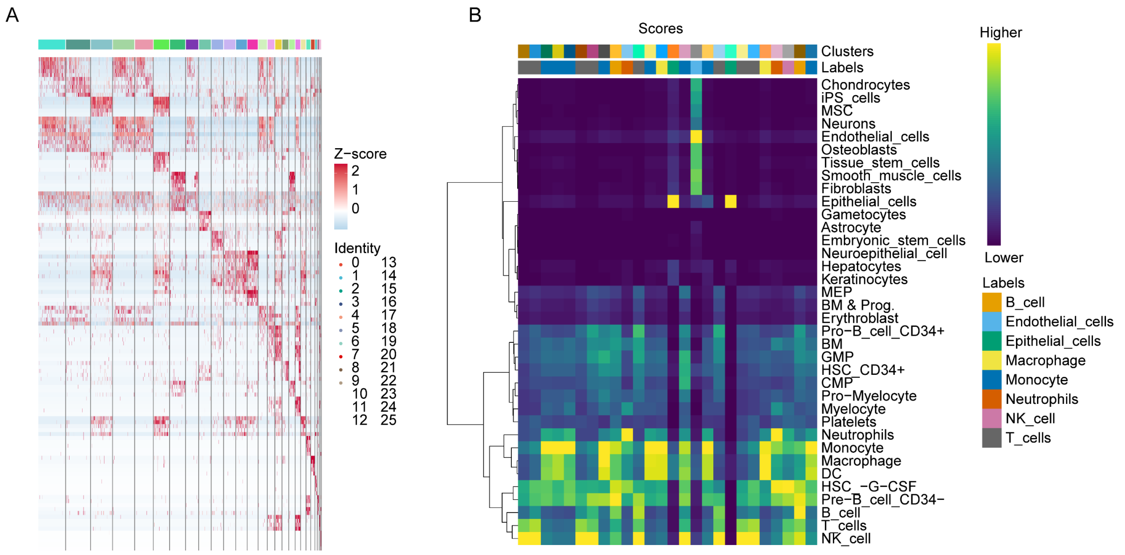

Supplement: Supplementary file 1 — Supplementary figures and tables. [file jcav16p1794s1.pdf]
